# Difficulties in Removing the Tracheotomy Tube after Diphtheria

**Published:** 1910-10-29

**Authors:** 


### Laryngology and Rhinology.

### DIFFICULTIES IN REMOVING THE TRACHEOTOMY TUBE AFTER DIPHTHERIA.

It sometimes happens that after tracheotomy has been performed on a child suffering from laryngeal diphtheria every attempt to remove the cannula is accompanied by recurrence of the dyspnœa even after the formation of membrane has long ceased; a similar difficulty also occurs when intubation has been performed. These cases have been variously ascribed to subglottic œdema, to paralysis of the adductors of the cords from disuse, and to spasm of the adductor muscles. When the tube cannot be removed after tracheotomy, the complication is a grave one; for, in view of the frequently associated bronchitis, the prolonged wearing of a cannula is fraught with serious danger in a young child debilitated by the primary disease. The causes of the difficulty are very various, and it is no easy matter to determine the source of the trouble in any individual case, for, if examination of the larynx is difficult in young children, still more so is that of the subglottic region and trachea.

### Spasm.

In a considerable proportion of cases the difficulty is due to psychical rather than to physical causes; nervousness and terror on the part of the little patient produce a spasm of the glottis as soon as the tube is taken out. The longer the cannula is left in the more likely is this to occur; it should, therefore, be removed as soon as possible after the formation of membrane has ceased, and, when antitoxin has been given early, it is seldom necessary to retain the tube for more than four days. A fenestrated tube should be worn and the external tube closed with a plug for some hours before removal. In cases managed in this way there is seldom any spasm when the tube is taken out. If dyspnœa does occur on removal of the tube, a fenestrated vulcanite tube should be inserted and the child encouraged to direct the air through the larynx by blowing soap bubbles, sounding a whistle, and talking, and a plug should cautiously be inserted from time to time without attracting the child's attention. The plan is sometimes successful of removing the tube and replacing only the flange. When a tube has to be worn for longer than a week or ten days the metal tube must be replaced by a vulcanite one. The greatest care must always be taken that the tube lies in the axis of the trachea. Parker's tubes are good or Durham's metal tube for low tracheotomies, but

Fuller's tubes, shaped in the segment of a circle, though easy to introduce, are very unsatisfactory. They pull forward the lower segment of the trachea, and so tend to form a kind of colotomy spur, and the tip of the tube impinges on the anterior tracheal wall. This has on several occasions been known to result in cicatricial stenosis, and the tip has even ulcerated into the innominate artery.

### STENOSIS.

Organic obstruction is most often due to subglottic œdema. This may follow a properly performed high tracheotomy, and it is therefore better when time allows to open the trachea fairly low down. But in the majority of cases it will be found that the operation, done in haste for urgent dyspnœa, has involved the larynx, that the cricoid has been divided and the tube inserted between its halves, or even that the tube has been thrust through the thyroid cartilage. In such cases a low tracheotomy should be performed and the upper wound allowed to close without further treatment, when the patency of the larynx will generally become re-established provided that the tube has not been kept in the upper wound too long. Occasionally the obstruction is due to kinking, or spur-formation, of the posterior wall of the trachea, the result of pulling forward the lower segment of the trachea by an imperfectly shaped tube, and sometimes to granulations about the tracheotomy wound projecting into the trachea. In such cases, again, the best treatment is to open the trachea lower down, insert a well-fitting tube, and so give complete rest to the original wound.

More active interference is rarely successful, though large masses of granulation tissue may be removed with curette or snare. Plugs extending upwards from the tracheotomy tube, and specially made T-shaped tubes, are badly borne and are unsatisfactory. A soft rubber tube passed into the trachea and held by a silk thread sewn through the tube and passed out through the tracheotomy wound is recommended by Killian and is less irritating. Prolonged intubation, if necessary with a specially long tube, has also given good results. However, as the stenosis has been caused by the irritation of a foreign body, it appears more rational to afford complete rest to the injured part by a low tracheotomy and to exercise great patience before attempting active dilatation. If necrosis of cartilage has occurred a very dense cicatricial stenosis results, which frequently defies all attempts at dilatation.

